# Filling In the Blanks on Solid Fuel Use: New Model Illustrates Trends, Highlights Needs

**DOI:** 10.1289/ehp.121-a227

**Published:** 2013-07-01

**Authors:** Julia R. Barrett

**Affiliations:** Julia R. Barrett, MS, ELS, a Madison, WI–based science writer and editor, has written for *EHP* since 1996. She is a member of the National Association of Science Writers and the Board of Editors in the Life Sciences.

Based on extensive data compiled by the World Health Organization (WHO), a newly developed model now reported in EHP illustrates country- and region-specific solid fuel use trends over the last 30 years, highlights data needs, and sets a course for improved assessment of solid fuel use as a global health indicator.^1^ Solid fuels used for cooking, such as wood, crop residues, dung, charcoal, and coal, generate household air pollution that is estimated to cause approximately 3.5 million premature deaths worldwide each year and contributes to respiratory and cardiovascular disease, cataracts, poor birth outcomes, and burn and scald injuries.^2^^,^^3^ Combustion products from cookstoves also increase outdoor air pollution, leading to an additional estimated 500,000 premature deaths annually.^2^^,^^3^ Women and children not only receive the greatest exposures to household air pollution but also often face additional hazards of injury or violence while collecting fuel.^3^^,^^4^

The WHO household energy database used as input for the model includes data from multiple international surveys on health, demography, and living standards; national censuses and surveys; and reports on poverty and the environment. “The main function of the database is to track trends in solid fuel use in different countries and different parts of the world so we can have a sense of whether we’re making any progress,” says Carlos Dora, a coordinator within the Department of Public Health and the Environment at the WHO, who was not involved in the study.

But one can’t simply look at the numbers in the database and draw solid conclusions about trends and needs. There are data gaps and fluctuations that must be accounted for, and if that’s not done appropriately via modeling, understanding may sharply deviate from reality. That would make for a shaky foundation for large-scale programs to improve environmental health.

To develop the current model, the researchers examined several statistical approaches and considered a range of covariates potentially associated with solid fuel use for cooking, including poverty level and urban versus rural population. They then tested the model by deliberately undercutting the source data according to three separate scenarios: omitting 20% of the data for countries with more than one survey, dropping the last survey for all countries with more than one survey, and removing 2008–2010 data from consideration. Under each scenario, the median difference between model estimates based on complete data and those based on a subset of data was less than 4%.

Next, using all available data for 150 countries in their model, the researchers estimated the proportion of households that used solid fuels for cooking from 1980 to 2010. Globally, the proportion dropped steadily from 62% to 41%, reflecting trends in every world region except sub-Saharan Africa, which at 77% in 2010 was only slightly lower than the 82% modeled for 1990. However, although proportions of solid-fuel users have declined, the world’s population has grown, and the absolute numbers of people using solid fuels for cooking has remained stable at approximately 2.8 billion since 1980. Furthermore, the minimal proportional decline in sub-Saharan Africa paired with the increased population meant nearly twice as many households used solid fuels for cooking in that region in 2010 as in 1980—646 million and 333 million, respectively.

**Figure:**
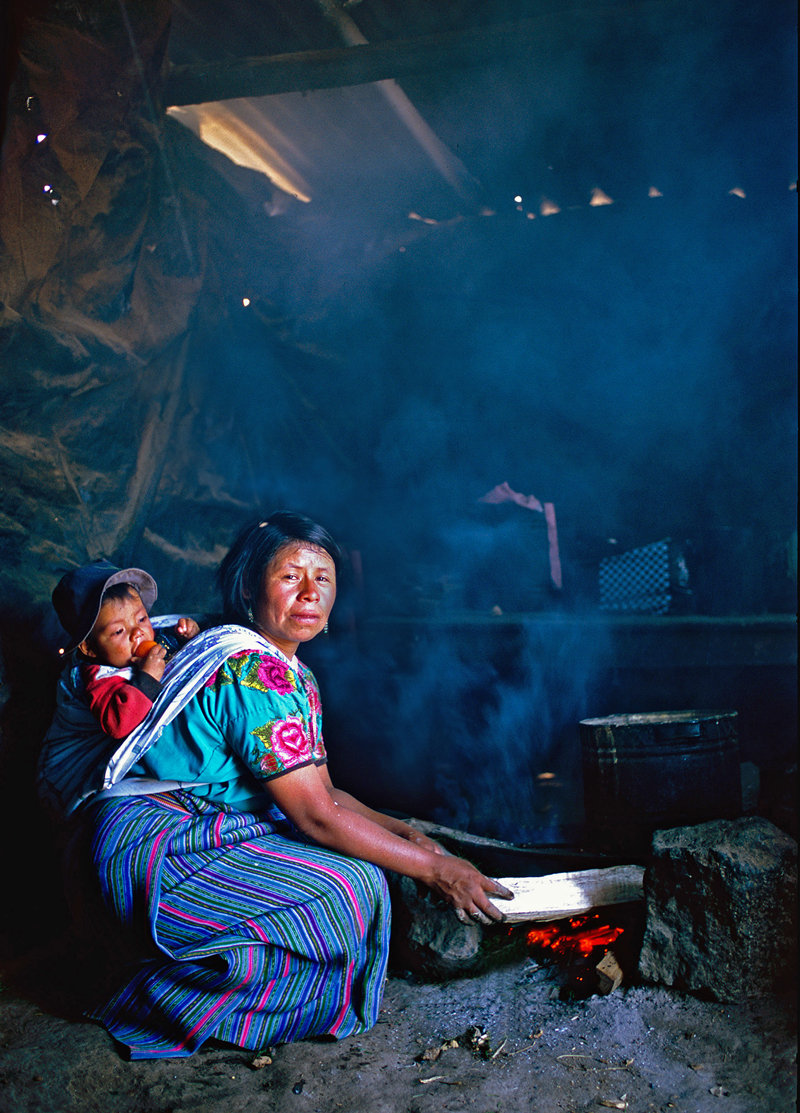
The proportion of solid-fuel users, like this family in Guatemala, has declined worldwide in recent decades, but the absolute number of users has remained stable at about 2.8 billion. Nigel G. Bruce/University of Liverpool

“There’s now a pretty good understanding of how much this database contributes—it’s the main source of information on cooking fuel, and it’s a very good database,” Dora says. “But we’re also aware that we should be improving as we move forward to understand the issues.” The current model is limited by the lack of data for factors that influence the amount and composition of household air pollution, such as stove and fuel type. Pollution from neighbors’ cookstoves and fuel used for heat and lighting also are not included.

Nevertheless, solid fuel use for cooking still serves as the single best indicator of exposure to household air pollution and the associated health effects.^1^ Additionally, the results are useful for endeavors such as the Global Alliance for Clean Cookstoves^5^ and the United Nations Secretary General’s Sustainable Energy for All initiative.^6^ “We’re at the moment when we’re discussing sustainable development goals, and it’s very likely that there’s going to be a similar development goal for energy, specifically access to clean and sustainable energy,” says Dora.

Meeting such goals will require a multidisciplinary approach, according to Daniel Kammen, director of the Renewable and Appropriate Energy Laboratory at the University of California, Berkeley. The databases, models, estimates, and various initiatives to deliver better technology are critical for reducing the hazards associated with household air pollution, he says. However, education, behavioral changes, and contributions from the social sciences are also part of the equation. “People think of hardware such as better technology and improved stoves, but there’s the human component, and that’s also important,” he says.
